# Integrating sustainability and least cost path analysis with a relative sustainability scoring index for optimal road planning

**DOI:** 10.1038/s41598-025-01030-1

**Published:** 2025-05-19

**Authors:** Ahmed K. Samy, Emad S. Bakhoum, Yasmeen A. S. Essawy, Khaled A. Hamdy

**Affiliations:** 1 Department of Structural Engineering, Ain Shams University (ASU), Cairo, Egypt; 2 Civil and Infrastructure Engineering and Management Department, Nile University, Giza, Egypt; 3 Civil Engineering Department, National Research Centre, Cairo, Egypt; 4 Department of Construction Engineering, The American University in Cairo (AUC), Cairo, Egypt

**Keywords:** Sustainability, Fuzzy logic theory, Sustainability index, Life cycle assessment, Multi criteria decision making, Sustainability, Civil engineering

## Abstract

Achieving sustainability in road construction is increasingly critical, yet traditional methods often focus solely on economic factors, creating a significant research gap in the holistic assessment of the three pillars of sustainability: economic, social, and environmental. The purpose of this research is to develop a ‘Relative Sustainability Scoring Index’ (RSSI) that addresses this gap. The research motivation stems from the need for a comprehensive tool that aligns with defined sustainability criteria. Therefore, it aims to quantitatively evaluate road projects, enabling a more robust understanding of the sustainability implications associated with various route planning strategies. The methods employed include the simple additive weight method, informed by expert input through fuzzy logic, and the application of Least Cost Path Analysis in QGIS to generate a suggested route. Civil 3D was utilized for road design and economic analysis, while VISSIM simulated traffic factors, and Simapro assessed environmental impacts. The results indicate that the suggested road achieved a sustainability score of 0.94, surpassing the present road’s score of 0.77. This research helps to improve the decision-making process by providing decision-makers with a quantitative tool that holistically evaluates roads based on economic viability, social acceptability, and environmental impact, thus advancing sustainable road network planning.

## Introduction

The concept of sustainability emerged several decades ago to integrate environmental, economic, and social considerations into project execution while minimizing detrimental effects on the environment. Over time, sustainability has become a fundamental principle guiding efforts toward achieving sustainable development. The construction sector plays a crucial role in advancing sustainable development by providing essential physical infrastructure to meet societal needs, however, it also poses significant negative environmental and social impacts. Moreover, conventional approaches to infrastructure project delivery are inherently unsustainable, as they heavily rely on natural resources and contribute to emissions, noise pollution, waste generation, habitat loss, and fragmentation. Despite their positive contributions to economic growth and regional development, these approaches ultimately impede sustainable development. Consequently, there is an increasing demand for and emphasis on integrating sustainability into infrastructure project delivery processes to enhance overall outcomes for sustainable development^1^. Furthermore, the sustainability of infrastructure projects significantly impacts the environment, economy, and society, as the processes involving the construction or enhancement of these projects can cause serious disruptions to human life and ecosystems. Additionally, challenges associated with infrastructure project development, such as time and cost constraints, may not always prioritize sustainability^2^. The construction of roads and highways plays a pivotal role in the socio-economic advancement of nations, serving as essential transportation infrastructure for both individuals and commodities. This significance is particularly evident in regions like the European Union, where roads serve as the primary means of passenger transportation and a vital conduit for freight. By connecting urban and rural areas, roads facilitate economic and social interactions, trade, and commerce, making them indispensable for societal development^3^. However, the sustainability of road infrastructure encompasses various complex aspects. While roads contribute positively to sustainability by improving market accessibility and fostering regional growth, investments in infrastructure are crucial for promoting economic expansion and addressing the financial deficits associated with highway construction.

The surge in infrastructure projects in Egypt, particularly those pertaining to road networks, underscores the urgent need for innovative solutions to establish a sustainable and efficient road network. A prevalent challenge in route design is the emphasis on the shortest path, often at the expense of exploring alternative options. This conventional approach, based on traditional grid leveling, frequently results in suboptimal solutions, leading to unnecessary costs and delays. Additionally, it introduces the potential for human error, which can significantly compromise the quality of road network design and planning. Furthermore, the selection of the route path often neglects the three pillars of sustainability (economic, social, and environmental), with decisions predominantly driven by economic criteria. This lack of holistic consideration hinders the development of a truly sustainable road network. Moreover, there is a conspicuous absence of comprehensive quantification and analysis of the factors encompassing these three pillars of sustainability in road planning and design; therefore, this research aims to address these gaps and contribute to the advancement of sustainable road network planning and design in Egypt.

The research question is how to plan and/or evaluate the roadway networks on a sustainability basis. Therefore, this research aims to develop a Relative Sustainability Scoring Index (RSSI) that aligns with defined sustainability criteria. This index will be created through a quantitative analysis of the factors within each sustainability pillar, and the integration of these quantitative assessments will enable a more comprehensive and robust understanding of the sustainability implications associated with different route planning strategies. By achieving this, the research can contribute to improving the decision-making process and facilitating the development of sustainable route networks that effectively balance economic, environmental, and social considerations. Additionally, this research makes significant contributions to the field of road construction sustainability, both theoretically and practically.

The theoretical contribution involves the development of the Relative Sustainability Scoring Index (RSSI), which offers a novel framework for systematically assessing road networks from a triple bottom-line sustainability perspective—economic, social, and environmental. This index allows decision-makers to evaluate the sustainability implications of various route planning strategies comprehensively. The practical contribution includes the use of least cost path analysis to optimize the route planning process and the utilization of Vissim and SimaPro for analyzing traffic behavior and environmental impact, respectively. By integrating these analytical tools with the developed RSSI, the research provides a robust methodology for evaluating road projects, ultimately leading to the development of more sustainable road infrastructure and contributing to the long-term well-being of both society and the environment.

## Literature review

Sustainable road construction is an essential component of modern efforts to align infrastructure development with environmental consciousness and long-term resilience. As the construction industry increasingly adopts innovative approaches to reduce its ecological footprint, road construction is undergoing a transformation toward more sustainable practices. A sustainable road infrastructure prioritizes minimizing environmental impact, conserving natural resources, and enhancing resilience, all while balancing transportation needs with ecological, social, and economic considerations. By integrating advanced technologies and eco-friendly materials, stakeholders can build roads that contribute to a circular economy, ensuring the well-being of both current and future generations.

In this context, a thorough literature review was conducted on sustainable road construction, primarily focusing on optimizing the route planning process. The review aimed to identify key factors that integrate economic, social, and environmental criteria, along with their respective impacts. By examining existing research, the goal was to understand how these sustainability factors influence road design and construction, ensuring that transportation infrastructure meets the needs of society while minimizing its ecological footprint and promoting long-term resilience.

### Optimization of route planning

Methods for planning linear infrastructures have advanced, with Geographic Information Systems (GIS) now making it easier to find suitable land corridors. One key technique in GIS is least-cost path analysis (LCPA), an optimization method that helps designers find the most cost-effective route between two points. LCPA works by considering factors like terrain, distance, and obstacles to minimize the overall cost or effort of the path. This method is widely used in areas such as transportation planning, environmental management, and urban development^4^.

Marjuki B. and Rudiarto I. in 2020 applied advanced spatial analysis techniques to optimize route planning for the Bawen-Yogyakarta Toll Road project^5^. Their research focused on integrating geotechnical and regional characteristics through methods such as Spatial Multi-Criteria Analysis (SMCA), Analytic Hierarchy Process (AHP), and Least Cost Path Analysis (LCPA). By considering factors such as land suitability, environmental risks, and infrastructure costs, they aimed to propose an alternative route that could be compared to the government’s preferred option. Their analysis demonstrated that the proposed route offered several advantages over the government’s route. These included better avoidance of high-risk areas such as those prone to earthquakes and landslides, improved preservation of protected areas, and more favorable topographical conditions with flatter terrain. Additionally, the alternative route was associated with lower construction costs due to fewer intersections with existing roads, rivers, and railways. Importantly, the route had a minimal negative impact on agricultural land, which is critical for the sustainability of the region. However, the proposed route had certain limitations. It was less effective in avoiding flood- and volcanic eruption-prone areas had higher land acquisition costs, and provided less support for industrial and tourism development. The study concluded that while the proposed route had clear advantages, further refinement of the analysis methods, data accuracy, and cost assessment strategies would be necessary to enhance the planning process and support regional infrastructure development more effectively.

Farooq et al. in 2021^6^ evaluated urban transport modes in Peshawar by collecting traveler preferences through a passenger survey and using GIS analysis to assess the spatial distribution of transport stops and population density. A SWOT analysis was performed to identify the strengths, weaknesses, opportunities, and threats for each mode, and multicriteria decision-making techniques—specifically the Best–Worst Method (BWM) and PROMETHEE II—were applied to rank the alternatives. The alternatives included a new Bus Rapid Transit (BRT) service, BRT with five additional stops, the existing old bus service (racket buses), wagon service, carpooling, and Careem/Uber. The results revealed that the BRT system with five additional stops was the optimal choice for Peshawar as it best met key criteria such as reliability, comfort, safety, and accessibility. The study concluded that this integrated methodology provided a robust framework for evaluating urban transport alternatives and could be adapted for similar applications in other cities. Similarly, Farooq et al. in 2019^7^ evaluated the transport plan for a high-speed railway connecting Beijing and Xiongan to determine the most suitable arrangement for passenger train stops. In the first stage, GIS and remote sensing techniques were employed to analyze settlement distribution, elevation, slope, vegetation, and route alignment, establishing the spatial context of the proposed railway. In the second stage, multicriteria decision-making methods—namely the Analytic Hierarchy Process (AHP) for weighting criteria and PROMETHEE II for ranking alternatives—were applied. The study considered alternatives with stops only in Beijing, a direct service with no stops, and configurations including stops in both Beijing and Guan, evaluating criteria such as travel time, number of stops, transport satisfaction, connectivity, operating costs, profit, and payback period. The results indicated that an alternative incorporating stops at both the distant metro station (Huangcun) and a station in Guan provided the best balance of economic and service-related factors. The study concluded that this integrated approach effectively supported decision-making by identifying a transport plan that was both economically viable and capable of enhancing connectivity and passenger satisfaction.

### Analyzing risks in road infrastructure projects

Effective risk management is recognized as essential for the success of road infrastructure projects, which are complex, costly, and critical to economic growth and public safety. Challenges such as budget overruns, delays, and operational inefficiencies are frequently encountered, jeopardizing project outcomes. In this context, Senic et al. in 2025^8^ observed that significant financial, technical, regulatory, and operational risks are faced by project managers, with over 90% of projects experiencing cost overruns that affect schedules and efficiency. A hybrid model was developed to assess risks across seven key categories—Design, External, Resource, Employer, Contractor, Engineer, and Project—using three main input factors: Environment, Contractual, and Design coefficients. Although various machine learning models were initially attempted, data limitations resulted in unsatisfactory predictions, and the use of fuzzy logic systems, particularly the Sugeno approach, was found to offer more accurate and reliable risk assessments. A systematic approach was provided for evaluating both major and secondary risks by combining empirical data, expert opinions, fuzzy logic, and result aggregation. Project complexity was defined through key coefficients, with fuzzy logic used to model the relationship between these coefficients and risk probabilities. Empirical probabilities of adverse events were integrated with fuzzy logic outputs to determine overall risk levels, and risks were prioritized based on their likelihood of occurrence. The hybrid model was found to effectively quantify risk probabilities by integrating project-specific characteristics, expert insights, and empirical data with fuzzy logic methods. Despite the challenges encountered by machine learning techniques due to limited data, the Sugeno fuzzy logic system demonstrated high accuracy and reliability, with deviations from real-world observations kept under 10%. This approach is beneficial in identifying critical risks, guiding targeted preventive measures, supporting better resource allocation, and improving project outcomes.

### Roads’ sustainability factors

Researchers have been focusing on identifying road sustainability factors. They extracted and prioritized key factors under each of the main sustainability pillars. A study developed an integrated index for evaluating highway sustainability based on the triple-bottom-line concept by Amr M. El‑Kholy et al. in 2020^9^. It analyzed 30 relevant studies on sustainability in project management, identifying 43 criteria contributing to the sustainability of highway projects. Another study conducted a comprehensive literature review by Ricardo Viana Vargas et al. in 2020^10^ to identify indicators for measuring the sustainability of infrastructure projects. It identified 97 potential indicators and validated 42 as essential. The study proposed a set of 21 new indicators that encompass the environmental, economic, and social dimensions. A different study by Mohamed O. Rageh et al. in 2023^11^ aimed to identify and analyze the factors that impact highway sustainability across the triple-bottom line of sustainability. It conducted a content analysis of published articles, SDGs, and infrastructure rating systems, and used Social Network Analysis to rank and prioritize 224 factors. The study identified 78 factors that had a significant impact on highway sustainability and found a lack of common factors addressing highway sustainability in widely used infrastructure rating systems. This underscored the need for a more comprehensive integration of sustainability factors in such rating systems.

### Developing a sustainability index and assessing road infrastructure

After identifying sustainability factors for road construction, the next step is to develop a sustainability index. Sustainable development has evolved to tackle global issues like climate change, resource depletion, and sociopolitical challenges, gaining momentum with the United Nations’ Sustainable Development Goals. However, road transport remains a major contributor to greenhouse gas emissions and other environmental and social concerns. Traditional road asset management systems focus on life cycle economic assessments, but to achieve sustainable outcomes, it is essential to also consider life cycle environmental and social impacts, evaluating emissions from roadworks and usage, as well as their effects on well-being.

Accordingly, Shafiq Alam et al. in 2017 proposed a new index called the “road sustainability index” (RSI), to deliver sustainable roadworks^12^. The RSI integrated key indicators from the economic, environmental, and social aspects of sustainability to achieve the best economic value, reduce GHG emissions, and improve social outcomes over the life cycle of road projects. It used data from conventional road asset management systems, making it a practical tool for improving road network sustainability. The study stressed the importance of reducing emissions, as road transport is a major contributor to climate change, and showed how the RSI could help manage environmental and social impacts. Examples of its use showed how it analyzed different road sections and treatment strategies, with economic and environmental factors affecting the results. The RSI proved useful for guiding decisions on sustainable road treatments and emphasized the need to include sustainability in road management practices. It also aligned with international goals, such as the UN Sustainable Development Goals, highlighting the need for innovation in sustainable infrastructure. Overall, the RSI was seen as a simple, effective tool to standardize sustainable road development, reduce costs and emissions, and address social impacts. It was ideal for managing new road projects, maintaining existing roads, and supporting global climate efforts.

## Research gap

The literature review highlighted the importance of sustainable road construction and identified three primary research areas: the development of alternative routes, the identification of sustainability factors, and the creation of a quantitative sustainability assessment tool for roads. While GIS software and least-cost path analysis were employed in studies to explore various route options, yielding positive results in economic feasibility, environmental sustainability, and social acceptability, there remained a notable research gap. Existing approaches often relied on traditional methods for route network design and exhibited limited use of least-cost path analysis, primarily focusing on economic or environmental factors while neglecting the integrated consideration of all three sustainability criteria. Furthermore, while progress was made in identifying and prioritizing key indicators within each sustainability pillar, there was insufficient quantitative analysis when considering all three pillars. Most studies leaned towards qualitative assessments, failing to thoroughly examine the quantification of factors within each sustainability dimension. This gap led to an incomplete understanding of the quantitative implications and impacts of various factors on sustainability. Additionally, current practices in route selection frequently prioritized economic considerations, undermining the holistic integration of economic, social, and environmental aspects crucial for sustainable road network development. This resulted in a lack of comprehensive quantification and analysis concerning the three sustainability pillars in road planning and design, particularly in the context of Egypt.

To address these gaps, this research aimed to develop a Relative Sustainability Scoring Index (RSSI) that evaluated road options based on economic viability, social acceptability, and environmental impact. By integrating the RSSI with least-cost path analysis, this study sought to provide decision-makers with a comprehensive tool for selecting the most sustainable road route among alternatives. Through this integrated approach, the research aspired to advance sustainable road network planning and design practices in Egypt, promoting a more balanced consideration of the three pillars of sustainability.

## Research methodology

**Fig. 1:**
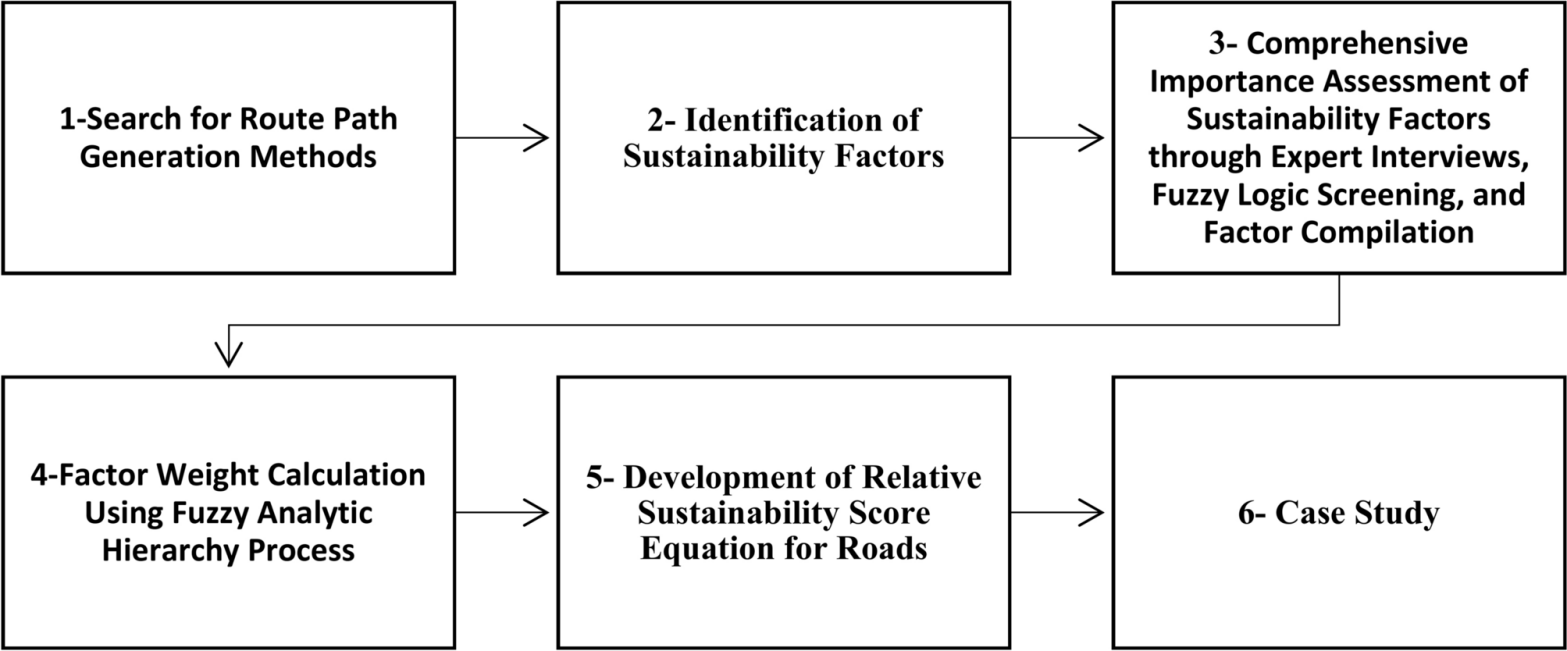
Research’s Framework.

The proposed relative sustainability scoring index was developed by the following steps shown in (Fig. 1):

1. Search for route path generation methods
  Conduct a comprehensive literature review to explore various methods and tools for generating route paths. This review led to the discovery of least cost path analysis, a widely used optimization method, within the QGIS software framework.
2. Identification of sustainability factors
  The identification of sustainability factors began with a comprehensive literature review on sustainability in highway construction, from which key economic and social aspects were extracted. This was followed by consultations with subject matter experts to assess the relevance of the identified factors and to introduce any overlooked considerations. This step was crucial to ensure that the developed Relative Sustainability Scoring Index (RSSI) effectively addressed the three pillars of sustainability—economic, social, and environmental. By incorporating expert opinions, the methodology enhanced the accuracy and contextual relevance of the sustainability factors, tailoring them to the specific challenges of road planning and construction in Egypt.
3. Comprehensive Importance assessment of sustainability factors through expert interviews, fuzzy logic screening, and factor compilation
  Experts in the field were interviewed to evaluate the significance of the sustainability factors identified through the literature review. To refine and prioritize these factors, triangular fuzzy logic was employed, as it effectively handled uncertainty in decision-making involving multiple, potentially conflicting factors. Fuzzy logic facilitated a trade-off between various factors, providing a more accurate representation of expert opinions, especially in cases of ambiguity or misunderstanding. This method, commonly used in construction management for multi-criteria decision-making, ensured a structured screening process and resulted in a comprehensive, well-vetted list of road sustainability factors.
4. Factor weight calculation using fuzzy analytic hierarchy process
  To allocate the relative importance of the identified sustainability factors, further expert interviews were conducted, followed by the application of the Fuzzy Analytic Hierarchy Process (FAHP). This method effectively integrated expert opinions to determine the weights of each factor, addressing the limitations of traditional AHP, which often required precise comparisons that were difficult to achieve due to the subjective nature of the criteria. FAHP enhanced flexibility by allowing decision-makers to use linguistic variables or fuzzy numbers, thereby managing uncertainties and enabling a more nuanced prioritization of sustainability factors. This approach ensured that each factor was appropriately weighted according to its significance in the context of sustainable road planning.
5. Development of relative sustainability score equation for roads
  A mathematical equation has been developed based on the simple additive weighting method to compute the relative sustainability score for roads, integrating the identified sustainability factors along with their corresponding weights.
6. Case study
  6.1 Alternative Route Generation Using QGI^13^
    The QGIS software was used for a least-cost path analysis. This method helped us create an alternative route that prioritized cost-effectiveness over distance. This approach allowed for a more detailed understanding of route optimization, moving beyond the simple shortest distance criterion, and considering a wider range of factors.
  6.2 Road design and economic analysis using Civil3D^14^
    The route design was done using Civil3D software, initially generated with QGIS. The design process followed AASHTO 2011 standards and considered the cadastral survey of the area. Crucially, the design process enabled the extraction of quantity takeoff data, which was key in examining the economic aspects of the roads. This provided a thorough understanding of the project’s cost-effectiveness and financial impact.
  6.3 Network analysis using VISSIM^15^
    VISSIM software was utilized for conducting network analysis. This powerful tool was specifically employed to compute various social factors, providing valuable insights into the social dynamics of the network.
  6.4 Environmental impact assessment with simapro^16^
    The study used Simapro software to thoroughly assess the environmental impact of a road construction project. This assessment covered all key stages of the project, including the construction phase with material transportation, and the operation phase. This holistic approach allowed for an in-depth understanding of the project’s environmental aspects, significantly enhancing the overall evaluation of the project’s environmental impact.
  6.5 Sustainability Evaluation of roads using the developed equation
    The developed sustainability formula was utilized to evaluate and compare the sustainability performance of two distinct roads. This method allowed for a direct comparison of the roads’ sustainability, providing valuable insights into their environmental, social, and economic impacts.

## Identification of sustainability factors

### Economic factors

Identifying the sustainability factors stood as a pivotal stage in devising the sustainability scoring formula, as these factors formed the foundation for decision-makers’ evaluations and aided in the decision-making process. To ensure thoroughness and relevance, the economic criteria, which were presented in (Table 1), were derived from an extensive review of the literature and interviews with experts in the field of road construction^17^. This approach ensured a comprehensive representation of the sustainability factors relevant to road construction.

**Table 1:** Economic sustainability factors.

Code	Criteria	This research	Reference										
			^9^	^18^	^19^	^20^	^21^	^22^	^23^	^24^	^10^	^25^	^26^
CC	Construction Cost	✓	✓	✓	✓	✓		✓	✓	✓			✓
MC	Maintenance Cost	✓	✓		✓	✓	✓		✓				
CT	Construction Time	✓					✓	✓	✓				
EP	Economic Performance	✓	✓	✓						✓			
ERC	Ecosystem Rehabilitation Cost	✓			✓	✓					✓	✓	✓

### Social factors

Similarly, the social criteria were also identified through the same rigorous process and were presented in (Table 2). This subsection specifically addressed the social implications of road construction, providing insights into how these factors influenced sustainability outcomes and decision-making processes.

**Table 2:** Social sustainability factors.

Code	Criteria	This research	References							
			^9^	^27^	^28^	^29^	^30^	^31^	^32^	^33^
TT	Travel Time	✓		✓	✓		✓	✓	✓	
SR	Safety of Roads	✓	✓	✓			✓			
TD	Travel Distance	✓						✓	✓	
AD	Average Delay	✓						✓	✓	
DVF	Daily Vehicle Flow	✓		✓			✓			
ADS	Average Driving Speed	✓					✓			
CH	Cultural Heritage	✓		✓	✓					✓
RG	Road Geometry	✓		✓					✓	
AC	Accessibility &Connectivity	✓		✓	✓				✓	
HR	Human Rights	✓				✓				✓
DE	Degree of Employment	✓	✓			✓				✓

### Environmental factors

The environmental factors influencing road construction are multifaceted, with each factor impacting a specific category. To thoroughly assess the environmental impact of road construction, it is essential to examine the entire process from raw material extraction to end use. In this context, the Recipe 2016 Endpoint (H) V1.08/World (2010) H/A methodology, implemented in Simapro software, is utilized. This methodology is a widely recognized approach for evaluating the environmental impacts of various processes^34^. It provides a comprehensive framework to quantify damage-oriented impacts across three major categories: human health, ecosystems, and resources. The methodology employs 22 impact categories as shown in (Table 3), each measuring specific aspects of damage, such as Disability-adjusted Life Years (DALY) for human health, species per year (species. yr) for ecosystems, and USD2013 for resources.

**Table 3:** Impact Categories of Recipe 2016 Method.

Human health “DALY”	Ecosystems “species.yr”	Resources “USD2013”
Global warming, Human health	Ozone formation, Terrestrial ecosystems	Mineral resource scarcity
Stratospheric ozone depletion	Terrestrial acidification	Fossil resource scarcity
Ionizing radiation	Freshwater eutrophication	
Ozone formation, Human health	Marine eutrophication	
Fine particulate matter formation	Terrestrial ecotoxicity	
Human carcinogenic toxicity	Freshwater ecotoxicity	
Human non-carcinogenic toxicity	Marine ecotoxicity	
Water consumption, Human health	Global warming, Terrestrial ecosystems	
	Land use	
	Global warming, Freshwater ecosystems	
	Water consumption, Terrestrial ecosystems	
	Water consumption, Aquatic ecosystems	

By offering valuable insights into the environmental consequences of different activities, the Recipe 2016 Endpoint methodology supports informed decision-making in environmental management and policymaking. Thus, it serves as a crucial tool in the study of environmental factors affecting sustainable road construction^35^.

## Assessment and prioritization of sustainability factors

### Experts interviews for evaluating sustainability factors’ importance

**Fig. 2:**
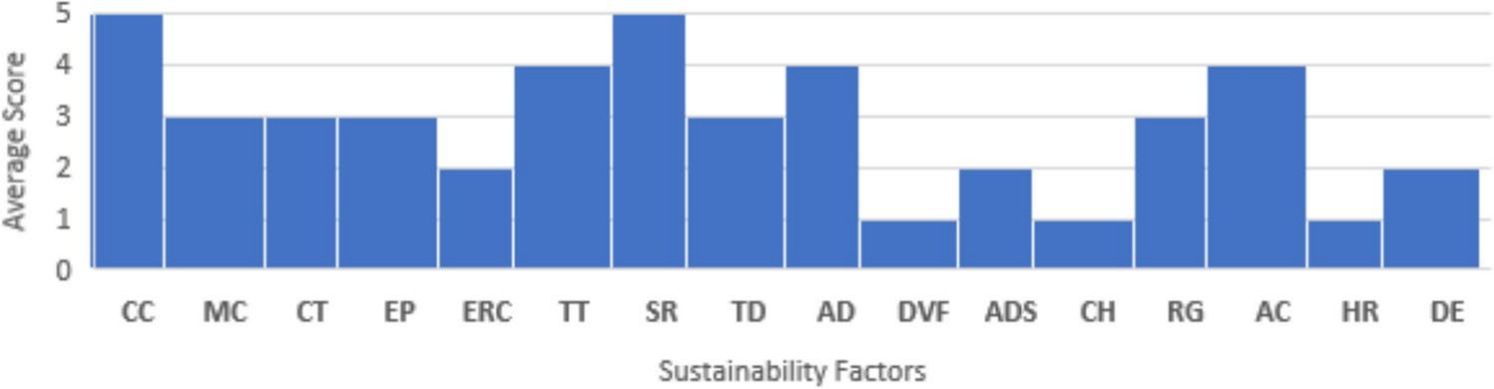
Average factors’ scores.

This study aimed to identify key factors crucial for sustainable development in highway projects. Interviews were conducted with experts in road construction, including project managers, consultants, designers, and a site engineer. These experts rated the importance of 16 identified factors on a Likert scale of 1 to 5, with 1 being extremely unimportant and 5 being extremely important. The average scores of the factors shown in (Fig. 2) were calculated based on the respondents’ answers, helping to understand the importance of each factor for sustainable development in highway projects. The main goal of these interviews was to screen the factors based on the experts’ responses, allowing for further analysis, and providing deeper insights into the factors that significantly impact the sustainable development of highway projects.

Cronbach’s alpha reliability assessment was performed to evaluate the consistency and reliability of the interview responses. The Cronbach’s alpha coefficient, which varies from 0 to 1, is considered acceptable if it exceeds 0.7, with values between 0.8 and 0.9 being particularly desirable^36^.The assessment was conducted on 16 questions, with the sum of all variances being 11.4 and the variance of the total score calculated as 35.84. The computed Cronbach’s alpha value was 0.73, indicating that the interview data are both reliable and valid for further analysis. This supports the subsequent exploration of significant findings related to the identification of socio-economic factors and the development of an index to quantify the sustainability performance of the highway construction project.

Cronbach’s alpha is calculated using the following formula:

1 $$\alpha = \frac{{\text{N}}}{{{\text{N}} - 1}}\left( {1 - \frac{{\sum \sigma_{i}^{2} }}{{\sigma_{{{\text{total}}}}^{2} }}} \right)$$

where N is the number of questions, $\sum \sigma _{i}^{2}$ is the sum of the variances for each individual question, $\sigma_{total}^{2}$ is the variance of the total score.

### Screening and prioritization of collected responses using triangular fuzzy logic methodology

The research utilized fuzzy sampling to address the limitations of conventional interview methodologies, enabling participants to express uncertainty in their responses. This approach yielded insights that aligned more closely with their actual thoughts, as fuzzy-based responses captured the subtleties of expert opinions and allowed for broader generalization, providing a more genuine representation than binary determinations^37^. Fuzzy logic membership functions, particularly triangular ones, were widely employed in construction management, especially in multi-criteria decision-making processes. Triangular fuzzy logic was chosen for its widespread acceptance and simplicity, making it one of the most utilized membership functions in fuzzy logic system design. This method effectively modeled complex decision-making processes involving imprecise or uncertain data, allowing for the integration of subjective human judgment. The linearity of the triangular membership function facilitated a clearer interpretation of results and enabled decision-makers to articulate varying degrees of membership, which was particularly crucial in assessing multiple, potentially conflicting sustainability factors in road planning. Overall, the application of triangular fuzzy logic enhanced the robustness and flexibility of the analysis, leading to a more comprehensive evaluation of sustainability criteria in the context of this research. Accordingly, the research used the fuzzy logic triangular membership function to prioritize, rank, and screen the identified sustainability criteria by the following steps^9,38^:

1. *Identify fuzzy numbers* Each linguistic term is represented by three numbers (l, m, u) which are the minimum, average, and maximum values of a criterion as shown in (Table 4).

**Table 4:** Fuzzy number for each linguistic term.

Linguistic term	Likert scale	Fuzzy number
Extremely unimportant	1	(0, 0, 0.25)
unimportant	2	(0, 0.25, 0.5)
moderately important	3	(0.25, 0.5, 0.75)
important	4	(0.5, 0.75, 1)
Extremely important	5	(0.75,1, 1)

2. *Calculate fuzzy weight numbers* This is done based on respondents’ views. The fuzzy weight number of a criterion is calculated using three values: the minimum number among all respondents, the average of all respondents, and the maximum number among all respondents. This is calculated using the following equations:
  The fuzzy weight number of criterion j, denoted as W_j_, is given by.
    2 $${\text{W}}_{{\text{j}}} = \left( {{\text{L}}_{{\text{j}}} ,{\text{M}}_{{\text{j}}} ,{\text{U}}_{{\text{j}}} } \right)$$
  The minimum number among all respondents, denoted as L_j_, is given by
    3 $${\text{L}}_{{\text{j}}} = {\text{min}}\left( {{\text{l}}_{{{\text{ij}}}} } \right)$$
  The arithmetic means of the average numbers among all respondents, denoted as M_j_, is given by
    4 $$M_{j} = \frac{1}{n}\mathop \sum \limits_{{i = 1}}^{n} m_{{ij}}$$
  The maximum number among all respondents, denoted as U_j_, is given by
    5 $${\text{U}}_{{\text{j}}} = {\text{max}}\left( {{\text{u}}_{{{\text{ij}}}} } \right)$$

here lij, mij, and uij represent the minimum, average, and maximum numbers of individual respondents i towards criterion j, respectively. i represents an individual respondent, j represents an identified criterion, and n is the number of respondents.

3. * Defuzzify the fuzzy weight numbers* This step converts the fuzzy weight numbers into crisp numbers for prioritizing and ranking. The crisp number after defuzzification, denoted as S_j_, is calculated using the equation.

6 $$S_{j} = \frac{{Lj + Mj + U_{j} }}{3}$$

4. *Set a threshold value* To filter criteria for inclusion in the sustainability index, a threshold value of 0.625 is set. This threshold is determined by averaging the maximum score of “moderately important” criteria (0.75) and the minimum score of “important” criteria (0.50), as recommended by the work done by Shu-Hsuan Chang et al. in 2010^39^. Criteria with a crisp number (denoted as S_j_) greater than or equal to 0.625 are deemed significant and included in the index. Conversely, criteria with a crisp number less than 0.625 are considered less significant and excluded from the index. This approach ensures that only the most relevant criteria contribute to the development of the sustainability index, aligning with established methodologies and recommendations. By adopting a threshold value derived from the arithmetic mean of importance scores, the process maintains consistency and ensures a balanced consideration of criteria in the index development process.

As demonstrated in (Table 5), the initial set of 16 factors was subjected to a comprehensive screening process. This resulted in a concise selection of 6 essential factors, which are Construction Cost, Travel Time, Safety of Roads, Travel Distance, Average Delay, and Accessibility and Connectivity. These will play a pivotal role in the formulation of the Relative Sustainability Score Index. These factors are designated for subsequent analysis to confirm their significance and contribution to the index.

**Table 5:** Screened sustainability factor list.

Code	Factor	L “Min”	M “Avg”	U “Max”	Crisp number	Status
CC	Construction Cost	0.5	0.925	1	0.81	Significant
MC	Maintenance Cost	0	0.425	1	0.48	less significant
CT	Construction Time	0	0.675	1	0.56	less significant
EP	Economic Performance	0	0.55	1	0.52	less significant
ERC	Ecosystem Rehabilitation Cost	0	0.1	0.75	0.28	less significant
TT	Travel Time	0.5	0.925	1	0.81	Significant
SR	Safety of Roads	0.25	0.85	1	0.70	Significant
TD	Travel Distance	0.25	0.75	1	0.67	Significant
AD	Average Delay	0.25	0.7	1	0.65	Significant
DVF	Daily Vehicle Flow	0	0.425	1	0.48	less significant
ADS	Average Driving Speed	0	0.325	1	0.44	less significant
CH	Cultural Heritage	0	0.15	0.75	0.30	less significant
RG	Road Geometry	0	0.575	1	0.53	less significant
AC	Accessibility and Connectivity	0.25	0.725	1	0.66	Significant
H R	Human Rights	0	0.1	0.75	0.28	less significant
DE	Degree of Employment	0	0.2	0.75	0.32	less significant

### Factor weight calculation using fuzzy analytic hierarchy process

After finalizing the sustainability factors, expert interviews were conducted to determine each factor’s importance using the Fuzzy Analytic Hierarchy Process (FAHP). Traditional AHP, a common method for estimating factor weights, relies on precise data, which can be challenging due to the subjective nature of expert judgments. To address this, Fuzzy AHP was used, which handles uncertain and subjective data more effectively by incorporating fuzzy set theory^40,41^. This allows decision-makers to convert vague expert input into specific decision intervals. Fuzzy AHP uses fuzzy numbers, which represent a range of possible values for a variable or rating, simplifying the representation of ambiguous linguistic ratings^42^. Triangular Fuzzy Numbers (TFNs), defined by the lowest possible, the modal (most likely), and the highest possible value, are commonly used in Fuzzy AHP^43^. This research utilized the tool developed by Morteza Dehghan Bahabadi in 2019^44^, which automates the process of collecting the experts’ answers based on their geometric mean on a fuzzy scale shown in (Table 6), evaluates the weight of each factor based on the FAHP method, and checks the consistency of the pairwise comparison matrix based on CRm which measures local consistency in pairwise comparisons, while CRg assesses overall consistency across the entire decision hierarchy. This tool is based on the work done by D.Y.Chang in 1996, Ozerk et al. in 1998, and Chia Chi Sun in 2010^45–47^

**Table 6:** Triangular membership function scale.

Fuzzy number	Linguistic term	Scale of fuzzy number
9	Perfect	(8, 9, 10)
8	Absolute	(7, 8, 9)
7	Very Good	(6, 7, 8)
6	Fairly Good	(5, 6, 7)
5	Good	(4, 5, 6)
4	Preferable	(3, 4, 5)
3	Not Bad	(2, 3, 4)
2	Weak Advantage	(1, 2, 3)
1	Equal	(1, 1, 1)

Following these interviews, the weight of each factor was determined. Additionally, the weight of each pillar of the triple-bottom-line was computed, as illustrated in (Table 7).

**Table 7:** Sustainability pillars and factors weights.

Factor	Local weight	Global weight	Consistency ratio (CRm)	Consistency ratio (CRg)	Consistency limit
Economic	0.45	0.45	0.021	0.0523	< 0.1
Social	0.34	0.34			
environmental	0.21	0.21			
Construction cost	0.45	0.45	–	–	–
Safety of roads	0.39	0.13	0.0143	0.0429	< 0.1
Travel time	0.23	0.08			
Accessibility and connectivity	0.16	0.05			
Average delay	0.12	0.04			
Travel distance	0.10	0.03			

## Development of relative sustainability score equation for roads

The proposed equation, as shown in Eq. 8, is based on the Simple Additive Weight (SAW) method, a common technique in decision-making. The SAW method sums up scores reflecting goal attainment under each criterion, multiplied by their respective weights^48^. This results in a weighted aggregate of performance ratings for each alternative or object across all criteria. The SAW method normalizes attribute ratings, allowing for a dimension-free evaluation. It integrates criterion values and weights to produce a unified value, emphasizing the maximization of evaluation criteria. However, it can also handle minimizing issues through specific formulas. Criteria subject to minimization or maximization are identified as costs and benefits, respectively^49^. In the context of sustainability factors, attributes like Construction Cost, Travel Time, Travel Distance, Average Delay, and Environmental Impact are defined as costs, while Safety of Roads and Accessibility and Connectivity are viewed as benefits. The SAW steps were implemented as outlined in previous studies by Hamed Taherdoost in 2023 and R A Surya et al. in 2019 was implemented as outlined below^50,51^

### Step 1: preparation of the decision matrix

Start by creating an initial matrix. This matrix helps organize information for m criteria and n alternatives or objects. Each element rij in the matrix represents the value of the i th criterion for the j th object. Where i = 1,2, 3,…,m and j = 1,2,3,….n

### Step 2: formulating the normalized matrix

Calculate the normalized value “rij” for each criterion of each alternative or object. This step considers whether the problem is focused on minimizing costs or maximizing benefits. As For cost-oriented factors, $rij = \frac{\min rij }{{{\text{rij}}}},$ while For benefit-oriented factors, $rij = \frac{{{\text{rij}}}}{{\max ri{\text{j}}}}$, where rij is the value of the i th criterion for j th alternative, the Max rij is the largest value of the i th criterion when all alternatives are compared, and the Min rij is the smallest value among them.

### Step 3: calculating the weighted normalized matrix

Combine the normalized criterion values with their respective weights to get a single performance value for each alternative.

7 $$Sj = \mathop \sum \limits_{i = 1}^{n} Wi*rij$$

Consequently, the relative sustainability scoring index “RSSI” for alternative i is defined as:

8 $$\begin{aligned} {\text{RSSI}} & { = 0}{\text{.45}}\frac{{{\text{Min of CC }}_{{\text{i to n}}} }}{{{\text{CCi}}}}{ + 0}{\text{.21}}\frac{{{\text{Min of EI}}_{{\text{ i to n}}} }}{{{\text{EIi}}}}{ + 0}{\text{.13}}\frac{{{\text{SR}}_{{\text{i}}} }}{{{\text{Max of SR }}_{{\text{i to n}}} { }}} \\ & \quad { + 0}{\text{.08}}\frac{{{\text{Min of TT }}_{{\text{i to n}}} }}{{{\text{TT}}_{{\text{i}}} }}{ + 0}{\text{.05}}\frac{{{\text{AC}}_{{\text{i}}} }}{{{\text{Max of AC}}_{{\text{ i to n}}} { }}}{ + 0}{\text{.04}}\frac{{{\text{Min of AD}}_{{\text{ i to n}}} }}{{{\text{AD}}_{{\text{i}}} }} \\ & \quad { + 0}{\text{.03}}\frac{{{\text{Min of TD}}_{{\text{ i to n}}} }}{{{\text{TD}}_{{\text{i}}} }} \\ \end{aligned}$$

where i denotes the specific alternative being evaluated, n represents the total number of alternatives being considered in the evaluation, CC_i_: Construction Cost of Alternative I, Min of CC_i to n_: Minimum value of the construction cost among the alternatives, EI_i_: Environmental Impact Score calculated from Simapro of Alternative I, Min of EI_i to n_: Minimum score of the environmental impact among the alternatives, SR_i_: Safety of Road Score of Alternative I, Max of SR_i to n_: Maximum score of the safety of road among the alternatives, TT_i_: Travel Time in Seconds calculated from VISSIM of Alternative I, Min of TT_i to n_: Minimum value of the travel time among the alternatives, AC_i_: Accessibility and Connectivity Score of Alternative I, Max of AC_i to n_: Maximum score of the accessibility and connectivity among the alternatives, AD_i_: Average Delay calculated from VISSIM of Alternative I, Min of AD_i_: Minimum value of the average delay among the alternatives, TD_i_: Travel Distance in meters calculated from VISSIM of Alternative I, Min of TD_i_: Minimum value of the travel distance among the alternatives.

## Case study

### Overview

**Fig. 3:**
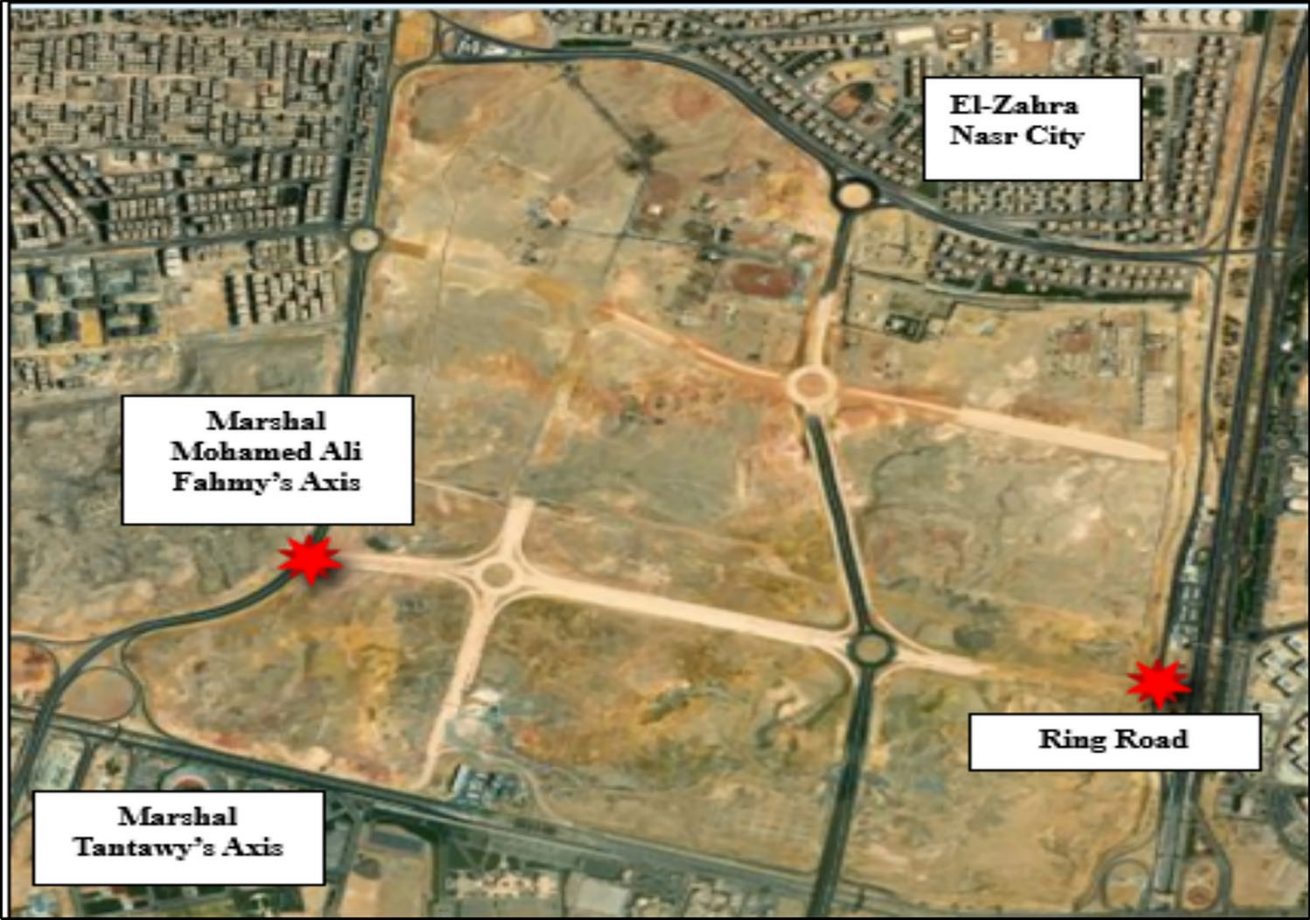
Site Layout by QGIS V.3.32.1^13^.

The case study focuses on sustainable route planning in an unpopulated area of about 6 Km2, in New Cairo, Egypt. The area presents challenges like extreme soil conditions with high slope variations and unsuitable soil composition of clay, organic materials, and loose sand. As part of an ongoing project, several internal roads are being developed. The focus is on the road connecting Marshal Mohamed Ali Fahmy axis with the ring road (Fig. 3). The goal is to apply sustainable route planning principles to identify and select the most sustainable road among alternatives, ensuring efficient transportation while minimizing ecological impact.

### Alternative route generation using QGIS

**Fig. 4:**
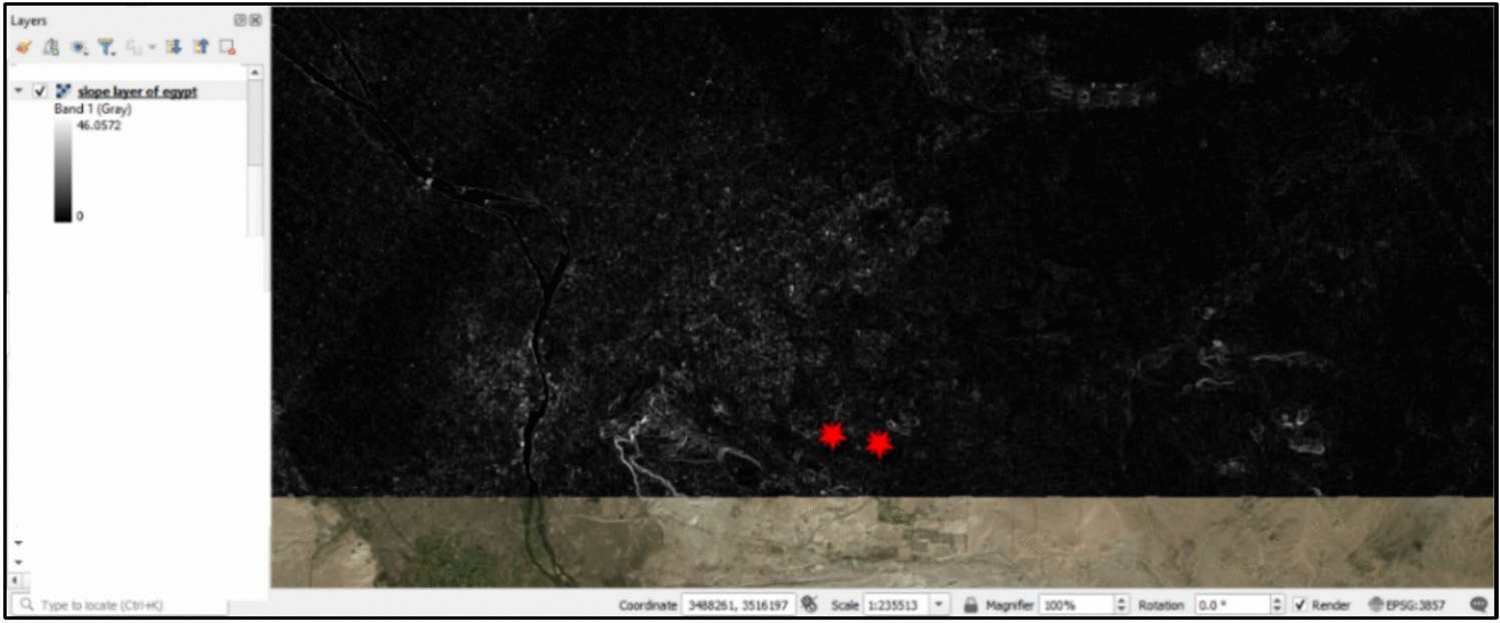
Slope Layer of Egypt by QGIS V.3.32.1^13^.

**Fig. 5:**
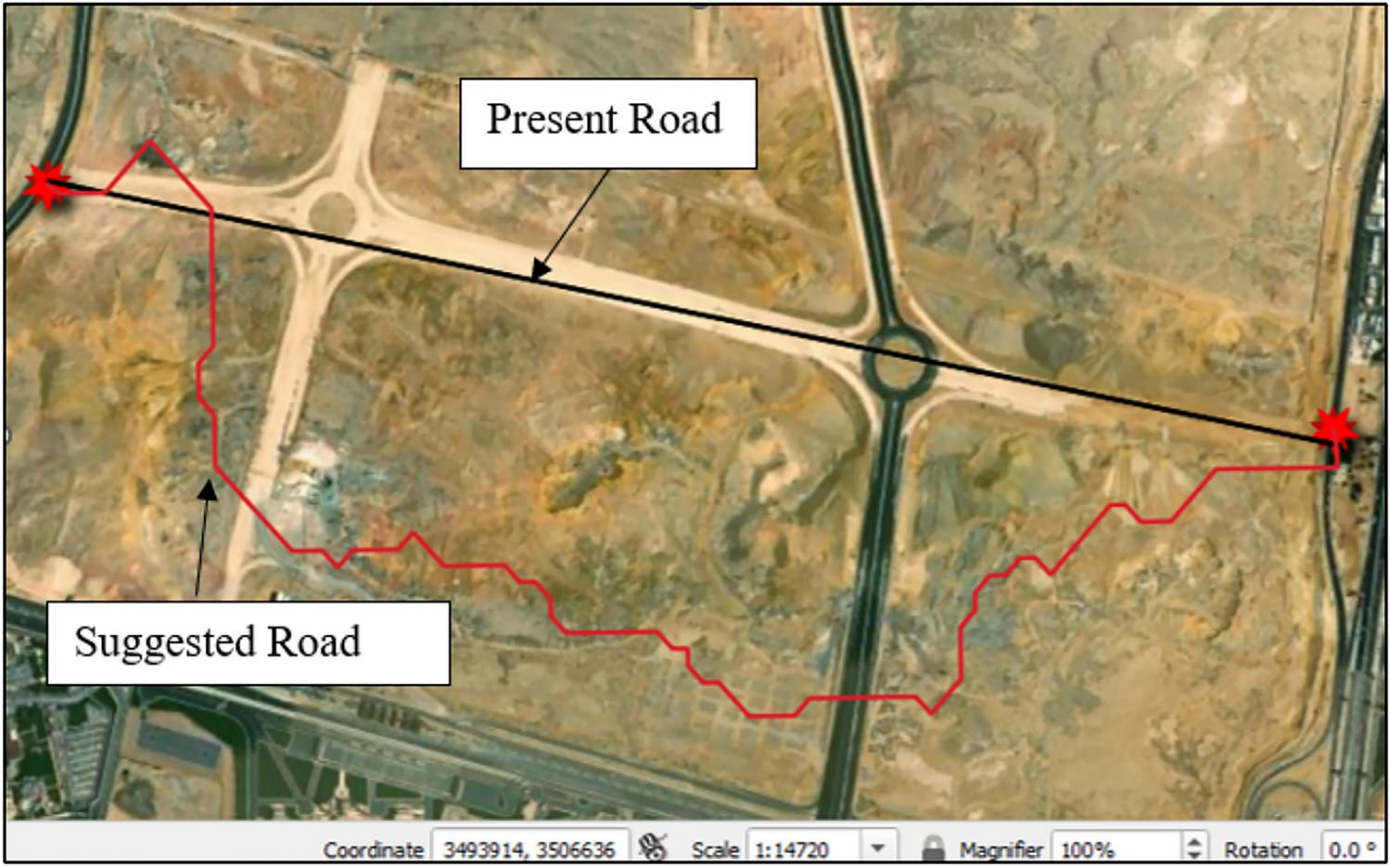
Present and Suggested Roads by QGIS V.3.32.1^13^.

In this case study, the initial route planning for the under-construction road, known as the “Present Road,” was based on the shortest distance between two points (Fig. 4). To explore alternatives, the study proposes Least Cost Path Analysis (LCPA) as an optimization method in transportation planning that aims to find the optimal path between two points while minimizing costs^52^. This analysis, integrated within QGIS software, uses the natural ground slope as the cost parameter. The goal is to find a route that reduces slope variations, resulting in a smoother, more direct path. To create the suggested road, the first step is to obtain a Digital Elevation Model (DEM), a matrix with elevation data for each grid, which can be downloaded directly from QGIS. Once the DEM is acquired, the cost raster is calculated, using the terrain slope as the cost value. The slope is calculated using QGIS’s slope algorithm, which assigns each cell a value between 0 and 90, indicating the slope degree between adjacent cells (Fig. 4). After calculating the slope values, the least cost path analysis is run, determining the optimal path based on the slope values. By using the least cost path code, the suggested road that minimizes slope variations is obtained (Fig. 5). This road provides a smooth route while also minimizing the distance. In summary, the process involves obtaining the DEM, calculating the slope using QGIS’s slope algorithm, and running the least cost path analysis to generate the suggested road^53^.

### Road design and economic analysis using Civil3D

**Fig. 6:**
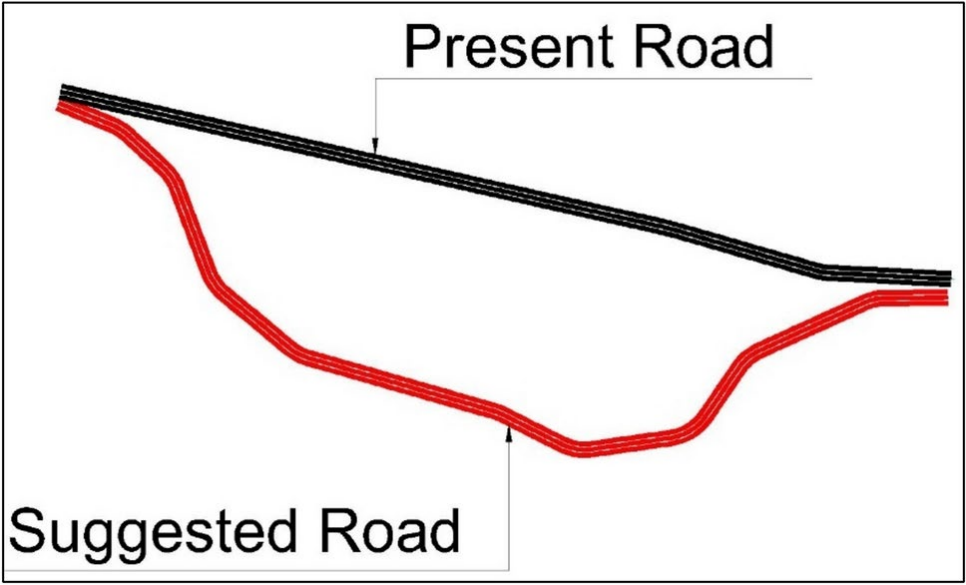
Present and Suggested Road Layout.

After getting the suggested route with its coordinates from the QGIS this route is to be designed by Civil3D software based on the AASHTO 2011 design method depending on the actual cadastral survey conducted in the area. It was found that this road with its current shape would not be safe as it has too many sharp edges and is not compatible with the design method, so a smoothed suggested road was made by drawing a strip of total width 80 m respecting the original route from the QGIS as shown in (Fig. 6).

**Fig. 7:**
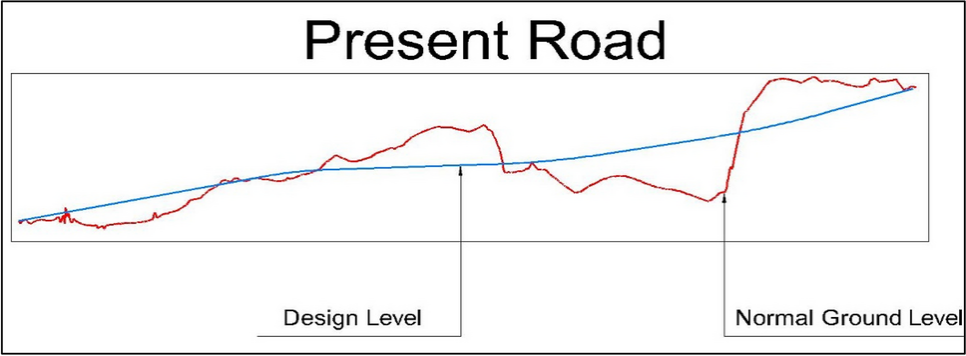
Present Road profile.

**Fig. 8:**
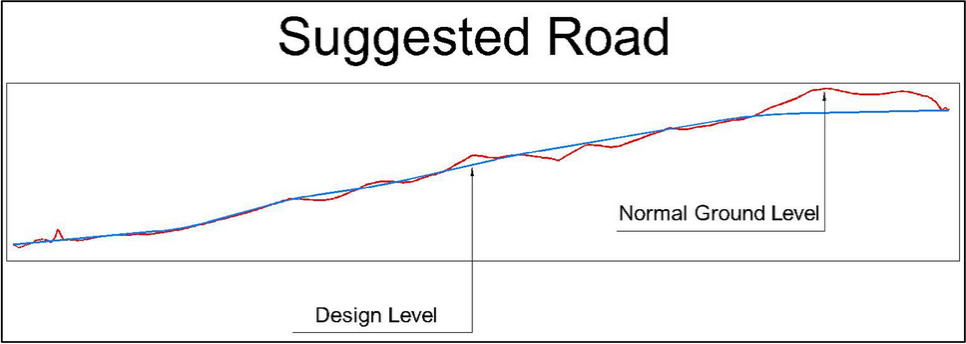
Suggested Road profile.

The proposed design involves building two roads, each with three lanes in both directions. Each lane will be 3.60 m wide, with safety shoulders of 0.60 m on each edge. The total asphalt width for each road will be 12 m per direction. The roads are designed for a speed of 60 km/hr. After design modifications, the present road is 2340 m long, while the suggested road is estimated to be 2770 m long. Detailed estimates for the quantities of cut and fill needed for road construction have been calculated. (Figs. 7 and 8) depict the road profiles, visually representing the design changes and the estimated quantities of cut and fill.

### Results

Based on the cut and fill quantities derived from Civil3D, by using the data in (Table 8),a detailed cost analysis is presented in (Table 9), illustrating the financial implications of the design modifications and variations in road length. It is crucial to note that these unit costs reflect the average rates in Egypt for 2023; consequently, the total construction costs for the present and suggested roads amount to EGP 73,998,690 and EGP 42,008,650, respectively.

**Table 8:** Road geometry.

Description	Unit	Value
Asphalt width	M	24
Granular subbase width	M	26
Subbase thickness	M	0.4
Asphalt thickness	M	0.12
Suggested road length	M	2770
Present road length	M	2340

**Table 9:** Present and suggested roads’ cost analysis.

No	Description of work	Unit	Present road quantities	Suggested road quantities	Unit Cost	Present road’s total cost	Suggested road’s total cost
1	Excavation in all types of soil except rocks and transporting the excavated material to a public waste dump	M^3^	399,795	177,950	42	EGP 16,791,390	EGP 7,473,900
2	Supplying & operating clean soil brought from outside	M^3^	348,180	21,750	85	EGP 29,595,300	EGP 1,848,750
3	Supplying & operating granular sub-base	M^3^	24,336	28,808	290	EGP 7,057,440	EGP 8,354,320
4	Providing bituminous layer “M.C.30” with a rate of 1.5 kg/m^2^	M^2^	56,160	66,480	29	EGP 1,628,640	EGP 1,927,920
5	Supplying & Operating Subbase Asphalt Layer with a thickness of 7 cm	M^2^	56,160	66,480	156	EGP 8,760,960	EGP 10,370,880
6	Providing Sticky bituminous layer “R.C.30” with rate 0.5 kg/m^2^	M^2^	56,160	66,480	12	EGP 673,920	EGP 797,760
7	Supplying & Operating Top Asphalt Layer with a thickness of 5 cm	M^2^	56,160	66,480	169	EGP 9,491,040	EGP 11,235,120
Total cost of structural work						EGP 73,998,690	EGP 42,008,650
Total cost of plumbing, electrical, and landscape work “Approximately 1,772,896.4 LE/Km”						EGP 4,148,578.61	EGP 4,910,924.25
Total construction cost						EGP 78,147,268.61	EGP 46,919,574.25

#### Network analysis using VISSIM

Transport modeling and simulation, essential for transport infrastructure planning and city logistics, involve tasks such as road network design, traffic analysis for congestion relief, delay reduction, and road safety improvement. Microscopic models, particularly PTV VISSIM software, are widely used for these tasks, modeling urban traffic flows, and promoting sustainable urban transportation and logistics. It enables the exploration of new logistics planning tools, simulates real city traffic, devises efficient traffic management strategies, and evaluates various intersection designs^54^. PTV VISSIM software is also used to estimate social sustainability factors, enhancing its utility in transport planning and urban development. Two models representing the present and suggested roads were developed based on road geometry and design velocity. The maximum flow capacity was determined using the theoretical maximum capacity equation^55^.

9 $$Qc = \frac{1000*V}{S}$$

where Qc: Maximum theoretical number of cars per lane, V: Velocity of the car in Km/hr, S: Average center-to-center spacing between cars = (0.2*V) + L, L: Average length of the cars in meters.

From the previous equation, we can estimate the number of cars by 11,057 Vehicles/hr.

From the VISSIM models, the following data per hour shown in (Table 10) were obtained:

**Table 10:** Network analysis for present and suggested roads.

Social factors	Present road	Suggested road
DELAYAVG(ALL)	7.93	15.70
Total distance in “meters”	4,967.11	5,157.55
Total travel time in “seconds”	338,906.18	395,957.65
Fuel consumption in “gallons”	115.01	148.62

#### Environmental impact assessment using simapro

The environmental impact of road construction and operation phases on human health, ecosystems, and resources was assessed using Simapro. This evaluation involved quantifying the amounts of sand, gravel, and bitumen as raw materials, as well as calculating the diesel consumption of construction equipment and the electric power usage of the asphalt batch plant. Additionally, fuel consumption by cars during the operation phase was considered. For the calculation of the quantities of raw materials, the density of limestone coarse aggregate and clean sand were taken by 2.2 and 1.8 ton/m3 respectively, in addition to the asphalt mix design was of 80% limestone coarse aggregate, 15% clean sand, and 5% bitumen, accordingly the following quantities shown in (Table 11) were calculated.

**Table 11:** Raw materials quantities.

Material	Unit	Present road	Suggested road
Limestone “CA”	Tons	66,208.90	78,375.49
Sand	Tons	629,099.57	41,962.10
Bitumen	Tons	791.86	937.37

**Fig. 9:**
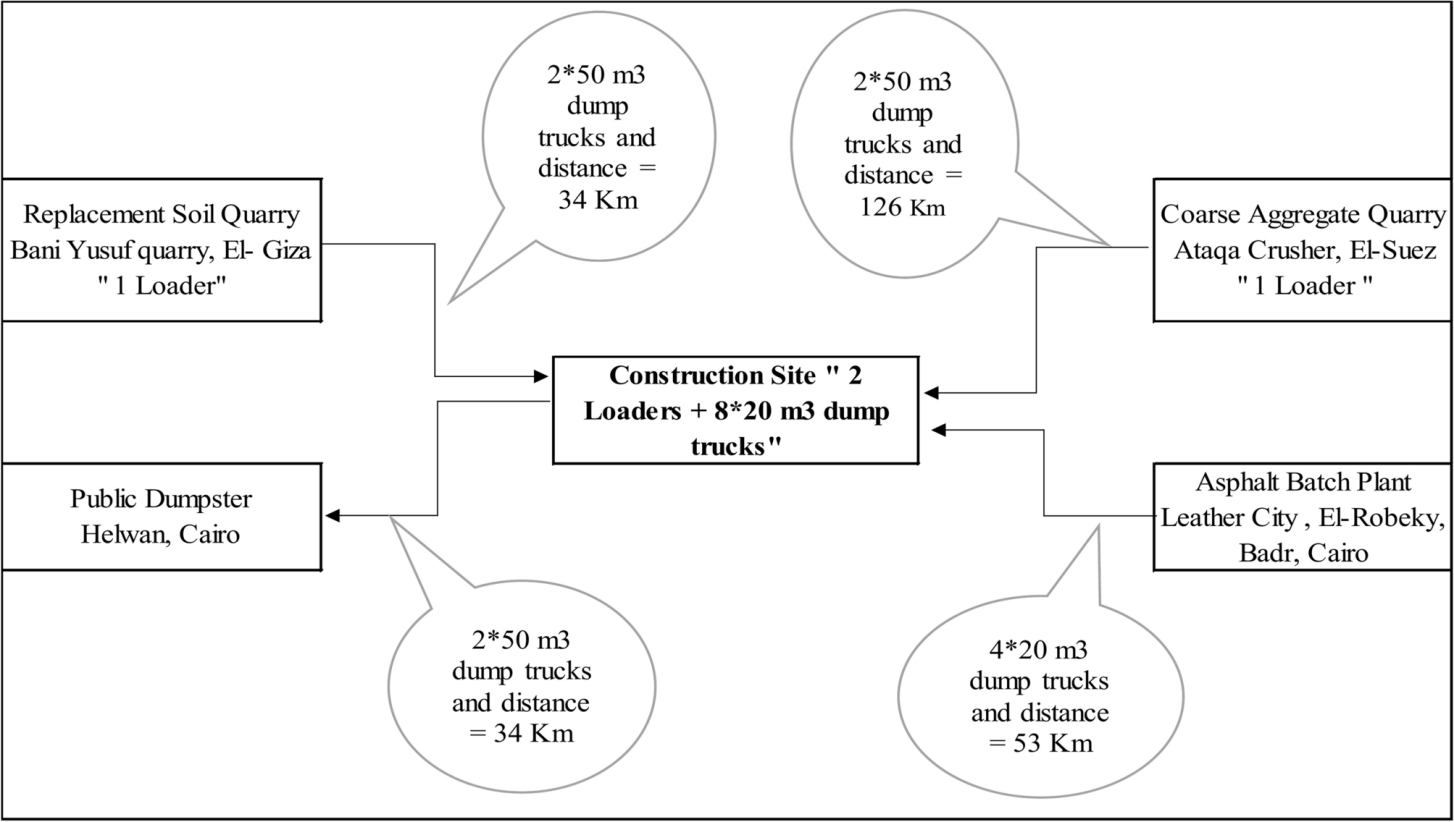
Raw material layout.

Diesel consumption of construction equipment was determined by calculating the construction process duration, transportation time for hauling materials, and time for material handling at the site. The transportation time analysis considered distances between raw materials mines and the site, and the number of equipment and trucks involved (Fig. 9). This data helped calculate the cycle time for each process and determine the production rate for hauling trucks. The production rate of the loading equipment was compared to the calculated production rate, and the lower value was selected as the process production rate (PR). (Table 12) shows the process of hauling construction waste from the site to public dumpsters.

**Table 12:** Production rate calculation for the excavated materials to the public dumpsters.

From site to dumpsters “helwan dumpster”		
Hauling distance	34	Km
PR of 1 loader	62.5	m^3^/hr
Truck capacity	50	m^3^
Hauling speed	50	km/hr
Return speed	75	km/hr
Loading time	0.4	hr
Unloading time	0.03	hr
Hauling time	0.68	hr
Return time	0.45	hr
maneuvering time	0.13	hr
Cycle Time	1.70	hr
PR of 1 truck	29.47	m^3^/hr
	235.76	m^3^/day
PR of 2 trucks	471.51	m^3^/day

The production rates for the construction equipment during the transportation process were obtained from the main contractor. By calculating the production rates, the corresponding durations of equipment operation were determined, as described in (Table 13). This table details the transportation time calculations for both the present and suggested roads.

**Table 13:** Present and Suggested Roads’ transportation time calculations.

Crew description	Unit	Number of crews	Production rate	Present road quantities	Suggested road quantities	Present road’s duration in “hrs”	Suggested road’s duration in “hrs”
2 Loader + 2*50 m3 Dump Trucks " for loading and unloading excavated materials to public dumpster"	m^3^/day	1	471.5	399,795	177,950	6783.2	3019.2
1 Loader + 2*50 m3 Dump Trucks " for loading Replacement Soil from quarry to site"	m^3^/day	1	381.6	348,180	21,750	7300.2	456.0
1 Loader + 2*50 m3 Dump Trucks " for loading Coarse Aggregate from Ataqa mine to site"	m^3^/day	1	154.9	24,336	28,808	1256.5	1487.5
1 Asphalt Batch Plant + 8*20 m3 Dump Trucks	m^3^/day	1	200	6,739	7,978	269.6	319.1
2 Loader + 8*20 m3 Dump Trucks “for loading and unloading materials within the site boundaries”	m^3^/day	1	1000	372,516	50,558	2980.1	404.5

In addition to transportation time, the construction time calculations for the present and suggested roads are presented in (Table 14). By analyzing the production rates for the construction processes, the durations of equipment operation were established.

**Table 14:** Present and suggested roads’ construction time calculations.

Crew description	Unit	Number of crews	Production rate	Present road quantities	Suggested road quantities	Present road’s duration in “hrs.”	Suggested road’s duration in “hrs.”
Bulldozer	m^3^/day	2	500	399,795	177,950	3198.4	1423.6
Grader “in wet soil with 25 cm thickness”	m^3^/day	2	500	348,180	21,750	2785.4	174.0
Grader “in subbase layers with 20 cm thickness”	m^3^/day	2	300	24,336	28,808	324.5	384.1
Compactor “in wet soil with 25 cm thickness”	m^3^/day	2	500	348,180	21,750	2785.4	174.0
Compactor “in subbase layers with 20 cm thickness”	m^3^/day	2	300	24,336	28,808	324.5	384.1
Screed asphalt paver	m^3^/day	1	200	6739	7978	269.6	319.1

Finally, the diesel consumption associated with the operation of construction equipment is illustrated in (Table 15). The total diesel consumption was calculated using the operation durations determined from both transportation and construction processes, with the diesel consumption per day provided by the main contractor.

**Table 15:** Present and Suggested roads’ total diesel consumption.

Equipment	Consumption rate “liters/day”	Present road		Suggested Road	
		total number of days	Diesel consumption “Liters”	Total number of days	Diesel consumption “liters”
Bulldozer	350	399.80	139,928	177.95	62,283
Grader “for soil”	240	348.18	83,563	21.75	5,220
Grader “for CA”	200	40.56	8,112	48.01	11,523
Loader	240	2,290.00	549,601	670.90	161,015
Asphalt batch plant	4000	33.70	134,784	39.89	159,552
Screed asphalt paver	192	33.70	6,470	39.89	7,658
Compactor	192	388.74	74,638	69.76	13,395
20 m^3^ dump truck	85	406.21	34,528	90.45	7,688
50 m^3^ dump truck	180	1,917.49	345,148	620.34	111,661
Total diesel consumption for construction and transportation processes “liters”			1,376,772		539,994

For the asphalt batch plant, the electric power consumption is estimated to be 2300 KWH, which is obtained from the operators in this plant. Based on the information presented in (Table 16), it can be inferred that the suggested road exhibits lower diesel consumption compared to the present road during the transportation and construction phases. This reduction can be attributed to a substantial decrease in earthwork quantities in the proposed road design, leading to more efficient resource utilization. While in the operation phase, the fuel consumption calculated from the VISSIM models as shown in (Table 16), assuming a 17-year service life of roads^56^, shows that the present road has a lower fuel consumption due to the length privilege.

**Table 16:** Present and suggested roads’ fuel consumption in the operation phase.

Fuel consumption in “liters”	Present road	Suggested road
	64,832,506.34	83,783,042.17

Simapro analysis provided data on raw materials quantities, diesel and fuel consumption for transportation, construction and operation phases, and power consumption of the asphalt batch plant. Results in (Table 17) show the present road is more environmentally friendly than the suggested road.

**Table 17:** Environmental impact single score for present and suggested roads.

Damage categories	Present road	Suggested road
Human health “DALY”	1.45	1.63
Ecosystems quality “species.yr”	0.0433	0.0465
Resource scarcity “USD2013”	0.139	0.173
Total score	1.63	1.85

#### Sustainability evaluation of roads using the developed equation

All sustainability factors were thoroughly analyzed, and the findings are presented in (Table 18). In addition to these factors, the scores of the safety of roads and accessibility and connectivity were assessed through the input of a focus group composed of four experts. Consensus was reached, indicating that the suggested road outperforms the present road in terms of safety, accessibility, and connectivity.

**Table 18:** Summarized results of the sustainability factors.

Alternatives	CC	EI	SR	TT	AC	AD	TD
Present road	78,147,268.61 EGP	1.63	3	338,906.18 Seconds	3	7.93	4,967.11 Meters
Suggested road	46,919,574.25 EGP	1.85	4	395,957.65 Seconds	5	15.7	5,157.55 Meters

Regarding road safety, the experts’ perspective highlighted that the presence of curves in the suggested road design plays a crucial role. These curves draw the driver’s attention, promoting adherence to speed limits and focus on the road. This aligns with a report from India in 2019, which revealed higher accident rates on straight roads compared to curved roads. Specifically, there were a total of 293,993 accidents recorded on straight roads, while curved roads accounted for 60,888 accidents^57^. In terms of accessibility and connectivity, the design of the suggested road holds a notable advantage. As the design of the land area is intended for a circular residential and commercial area, a curved road layout facilitates improved accessibility and connectivity within the community. Therefore, considering the insights from the focus group and the evidence regarding safety and accessibility, the suggested road is deemed superior in these aspects.

Utilizing the Relative Sustainability Scoring Index (RSSI) developed from Eq. 8 and the relevant data presented in (Table 18), the suggested road achieved a sustainability score of 0.94, significantly higher than the present road’s score of 0.77, indicating a lower level of sustainability. This evaluation clearly identified the suggested road as the more sustainable alternative.

#### Validation through sensitivity analysis of criteria weighting

Sensitivity analysis is widely recognized as a pivotal component of multicriteria decision-making (MCDM). It evaluates how changes to input parameters, such as criteria weights, impact the outcomes of a decision-making model by systematically varying these parameters across their feasible ranges. This process is invaluable because the results produced by MCDM models often depend heavily on input data, which can be influenced by factors like personal opinions, cognitive biases, and measurement errors. By identifying the variables that exert the most substantial influence over model outputs, sensitivity analysis enables decision-makers to refine the precision of these critical factors and better understand the resilience of the model. Ultimately, this leads to more reliable and effective decisions. In practical applications, altering the prioritization of criteria and the selected MCDM methods can lead to changes in the outcomes. Such variations are used not only to validate the robustness of the model but also to provide insights into how different weighting schemes influence alternative rankings^58^.

Following the methodology of Pamucar et al. 2017^59^, A sensitivity analysis was performed in this study to assess how changes in the weights assigned to the criteria would affect the ranking of the alternatives. Sensitivity analysis was conducted through 11 scenarios (Table 19), which illustrate the impact of favoring certain criteria over others. The first eight scenarios were designed to prioritize each individual criterion by assigning it a higher weight while equally distributing the remaining weight among the other factors. The last three scenarios incorporated combinations of priorities, specifically focusing on the social criteria factors. The basis for selecting these combinations and determining the weights was expert input and insights from previous studies^60^. This comprehensive approach ensured that the weight coefficients accurately reflected real-world conditions and allowed for a thorough evaluation of how variations in criteria prioritization influenced the final alternative rankings.

**Table 19:** Alternative selection scenarios under varying criteria weights and preferences.

Scenarios “SC”	Sustainability factors						
	CC	EI	SR	TT	AC	AD	TD
SC1-uniform weight criteria	0.143	0.143	0.143	0.143	0.143	0.143	0.143
SC2-priority of CC	0.4	0.1	0.1	0.1	0.1	0.1	0.1
SC3-priority of EI	0.1	0.4	0.1	0.1	0.1	0.1	0.1
SC4-priority of SR	0.1	0.1	0.4	0.1	0.1	0.1	0.1
SC5-priority of TT	0.1	0.1	0.1	0.4	0.1	0.1	0.1
SC6-priority of AC	0.1	0.1	0.1	0.1	0.4	0.1	0.1
SC7-priority of AD	0.1	0.1	0.1	0.1	0.1	0.4	0.1
SC8-priority of TD	0.1	0.1	0.1	0.1	0.1	0.1	0.4
SC9-priority of SR & TT	0.08	0.08	0.3	0.3	0.08	0.08	0.08
SC10-priority of AC & AD	0.08	0.08	0.08	0.08	0.3	0.3	0.08
SC11-priority of AD & TD	0.08	0.08	0.08	0.08	0.08	0.3	0.3

The corresponding scores and rankings of the alternatives by scenarios are shown in (Tables 20 and 21). The results demonstrated that assigning different weights to the criteria leads to changes in the ranking of alternatives, confirming the sensitivity of the methods to variations in the weight coefficients. This validation step enhances the robustness and generalizability of the model’s results.

**Table 20:** Alternative scoring under diverse criteria weighting scenarios.

Alternative	Scenarios										
	SC1	SC2	SC3	SC4	SC5	SC6	SC7	SC8	SC9	SC10	SC11
Present road	0.85	0.78	0.90	0.78	0.90	0.82	0.90	0.90	0.83	0.86	0.92
suggested road	0.89	0.92	0.89	0.92	0.88	0.92	0.77	0.91	0.90	0.83	0.82

**Table 21:** Ranking of alternatives across various criteria weighting scenarios.

Alternative	Scenarios										
	SC1	SC2	SC3	SC4	SC5	SC6	SC7	SC8	SC9	SC10	SC11
Present road	2	2	1	2	1	2	1	2	2	1	1
Suggested road	1	1	2	1	2	1	2	1	1	2	2

## Analysis and implications

This research provided a comprehensive analysis of the three pillars of sustainability: economic, social, and environmental. The analysis focused on the construction phase concerning economic criteria, the operational phase regarding social criteria, and encompassed the entire lifecycle of environmental criteria, from raw material extraction through to transportation, construction, and end-use.

**Fig. 10:**
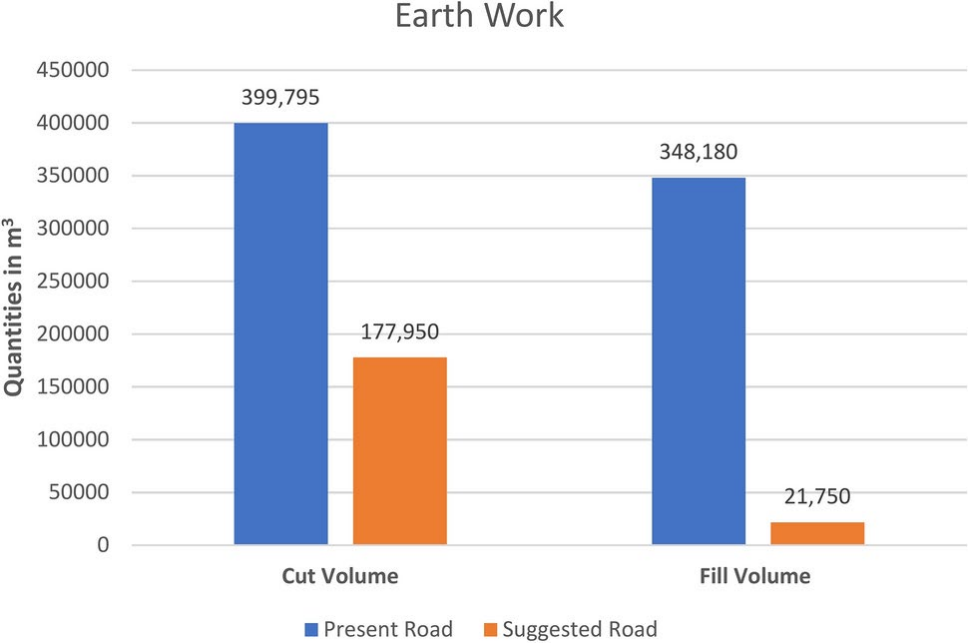
EarthWork for the two roads.

Utilizing the developed Relative Sustainability Scoring Index (RSSI) as outlined in Eq. 8, the sustainability of both the present and suggested road alternatives was evaluated, yielding scores of 0.77 and 0.94, respectively. The higher score of the suggested road indicated its superior overall sustainability, particularly concerning cost, safety, and accessibility. Specifically, the suggested road demonstrated a notable advantage in construction costs, amounting to EGP 46,919,574.25 compared to EGP 78,147,268.61 for the present road. The analysis revealed that the suggested road aligned more favorably with the natural ground level than the present road, resulting in reduced quantities of cut and fill (Fig. 10). Consequently, this alignment led to lower earthwork costs. However, it was important to note that the suggested road was approximately 430 m longer than the existing one, necessitating additional subbase materials, asphalt, and other non-structural elements, which contributed to increased costs.

Based on the cost analysis, the research concluded that the suggested road offered significant economic advantages compared to the present road, achieving a savings of 40% in construction costs.

Conversely, the present road excelled in social and environmental dimensions. In terms of social factors, the present road outperformed the suggested alternative across all categories, particularly in travel time (338,906.18 s vs. 395,957.65 s), average delay (7.93 s vs. 15.7 s), and travel distance (4,967.11 m vs. 5,157.55 m). The present road’s shorter total length contributed to its favorable performance in network analysis.

**Fig. 11:**
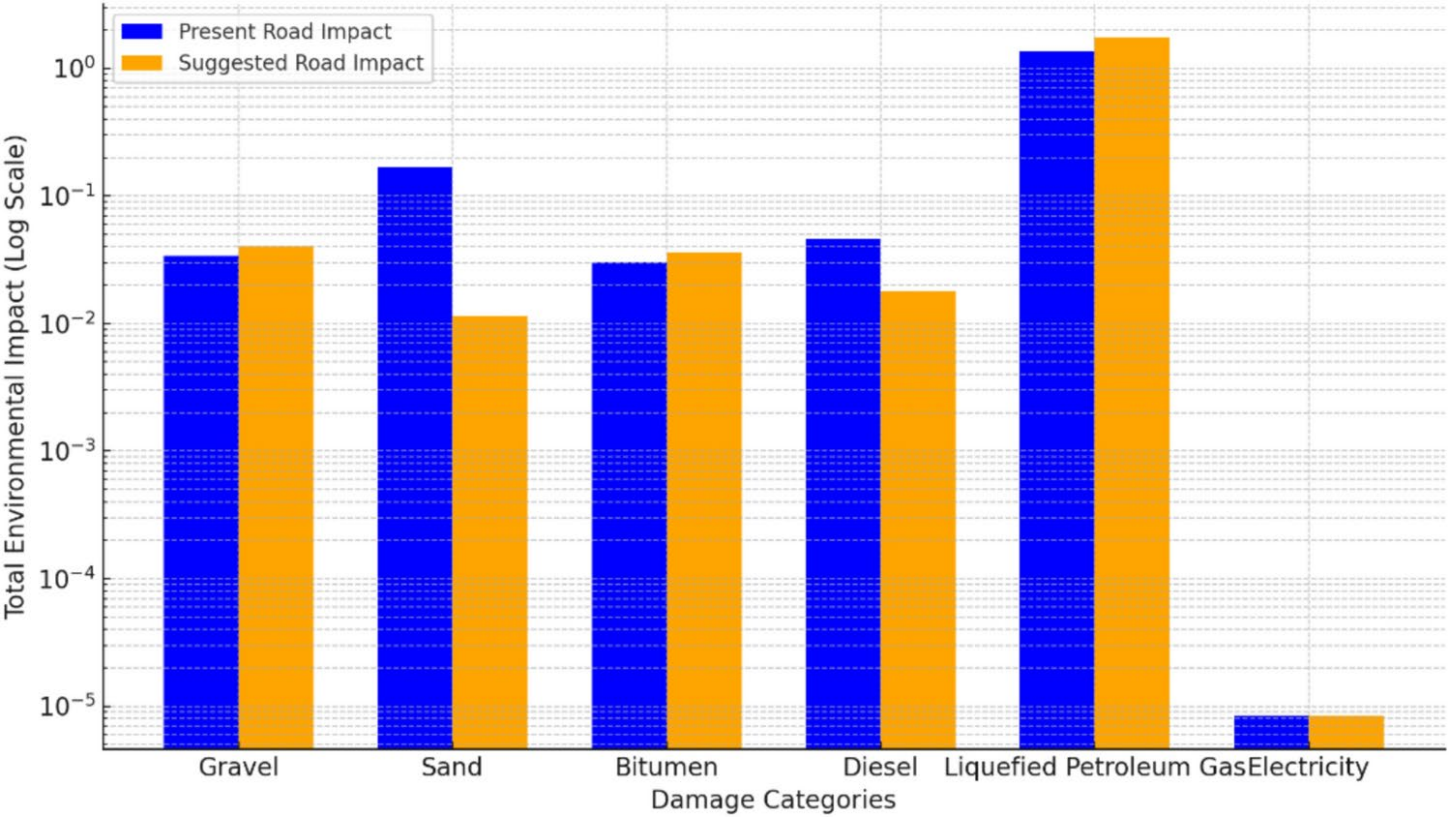
Comparative Analysis of Present Versus Suggested Road Impact.

From an environmental standpoint, the present road exhibited a lower overall impact score (1.63 vs. 1.85), making it more eco-friendly than the suggested road. However, as illustrated in (Fig. 11), the suggested road demonstrated better environmental performance regarding sand and diesel emissions.

Despite the present road’s advantages in social and environmental aspects, the significant improvements in cost, safety, and accessibility of the suggested road contributed to its higher overall sustainability score. This comparative analysis underscored the effectiveness of the RSSI formula in providing a holistic assessment of sustainability by integrating economic, environmental, and social dimensions. This comprehensive approach empowered decision-makers to make informed choices, positioning the suggested road as the preferred option for future development initiatives.

## Conclusions and recommendations

In conclusion, this research presented a novel and practical framework for addressing the complex challenges of road construction sustainability. By integrating the Least Cost Path Analysis and the Relative Sustainability Scoring Index, decision-makers and stakeholders in road construction can make informed choices during the planning and design phase, promoting sustainable practices in the field. Through a comprehensive literature review and expert consultations, a set of six critical factors for road sustainability was identified and weighted using the Fuzzy Analytic Hierarchy Process. The developed Relative Sustainability Scoring Index proved to be an effective tool for assessing and comparing the sustainability of different roads, additionally, the RSSI Equation can be applied across different geographic contexts; however, the case study was conducted in Egypt to take advantage of reliable and actual site data.

The case study conducted in this research validated the proposed methodology, demonstrating its efficacy in identifying the optimum sustainable road solution. The suggested road exhibited a significantly higher sustainability score (0.94) compared to the present road (0.77), highlighting its superior overall performance in economic, social, and environmental aspects.

In summary, this research provides a valuable contribution to the field of road construction sustainability by offering a practical framework that empowers decision-makers to balance socioeconomic development with environmental preservation. By adopting this integrated approach, the negative impacts of roads can be mitigated, leading to more sustainable road infrastructure, and contributing to the long-term well-being of both society and the environment.

It is worth noting that, future research should focus on evaluating the sustainability of entire road networks, including bridges, tunnels, and roundabouts, to better account for the interconnectedness of infrastructure and the cumulative environmental effects. Additionally, studying the influence of road signals, particularly the impact on waiting times, can provide insights into traffic efficiency and emissions reduction. Exploring ways to improve sustainability during the operational phase, such as using rainwater for maintenance or integrating smart technologies like nanoparticle-infused asphalt to generate electricity, could significantly enhance road performance and sustainability. Furthermore, expanding the scope of sustainability assessments to encompass inhabited areas will result in a more comprehensive understanding of the long-term environmental, social, and economic impacts of road construction, thereby leading to more informed decision-making processes in the future.

## Data Availability

The datasets used and/or analysed during the current study available from the corresponding author on reasonable request.
